# Safety and Yield of Diagnostic ERCP in Liver Transplant Patients with Abnormal Liver Function Tests

**DOI:** 10.1155/2014/314927

**Published:** 2014-07-09

**Authors:** Jayapal Ramesh, Nipun Reddy, Hwasoon Kim, Klaus Mönkemüller, Shyam Varadarajulu, Brendan McGuire, Derek DuBay, Devin Eckhoff, C. Mel Wilcox

**Affiliations:** ^1^Division of Gastroenterology and Hepatology, Endoscopic Ultrasonography, Basil Hirschowitz Endoscopic Center of Excellence, University of Alabama at Birmingham, BDB 389, 1808 7th Avenue South, Birmingham, AL 35294, USA; ^2^Division of Gastroenterology and Hepatology, University of Alabama at Birmingham, Birmingham, AL 32803, USA; ^3^Center for Interventional Endoscopy, Florida Hospital, Orlando, FL 35294, USA; ^4^Department of Liver Transplantation Surgery, University of Alabama at Birmingham, Birmingham, AL 32803, USA

## Abstract

*Background*. Abnormal liver enzymes postorthotopic liver transplant (OLT) may indicate significant biliary pathology or organ rejection. There is very little known in the literature regarding the current role of diagnostic ERCP in this scenario. *Aim*. To review the utility of diagnostic ERCP in patients presenting with abnormal liver function tests in the setting of OLT. *Methods*. A retrospective review of diagnostic ERCPs in patients with OLT from 2002 to 2013 from a prospectively maintained, IRB approved database. *Results*. Of the 474 ERCPs performed in OLT patients, 210 (44.3%; 95% CI 39.8–48.8) were performed for abnormal liver function tests during the study period. Majority of patients were Caucasian (83.8%), male (62.4%) with median age of 55 years (IQR 48–62 years). Biliary cannulation was successful in 99.6% of cases and findings included stricture in 45 (21.4 %); biliary stones/sludge in 23 (11%); biliary dilation alone in 31 (14.8%); and normal in 91 (43.3%). Three (1.4%) patients developed mild, self-limiting pancreatitis; one patient (0.5%) developed cholangitis and two (1%) had postsphincterotomy bleeding. Multivariate analyses showed significant association between dilated ducts on imaging with a therapeutic outcome. *Conclusion*. Diagnostic ERCP in OLT patients presenting with liver function test abnormalities is safe and frequently therapeutic.

## 1. Introduction

Complications after orthotopic liver transplantation (OLT) include allograft rejection, infections due to immunosuppression, disease recurrence, and biliary tract pathology. Amongst these, biliary tract disease remains the most common, identified in up to 40% of cases [1–3]. The majority of these biliary disorders respond well to endoscopic management and prompt therapy avoids graft dysfunction and results in good outcomes [4]. Furthermore, self-expanding metal stent insertion for treatment of biliary complications after liver transplant is an alternative to surgery [5, 6]. However, diagnosing biliary complications after liver transplant is challenging as presentation can be atypical with nonspecific symptoms while laboratory and imaging testing are poorly sensitive [7, 8]. The algorithm employed to investigate includes transabdominal ultrasound scan with Doppler studies followed by MRCP and endoscopic or percutaneous cholangiography for therapy depending on the anatomy. Although ERCP with dynamic cholangiography is considered the gold standard for investigating the biliary tree, the procedure is invasive and has now transitioned primarily into a therapeutic endeavor due to evolution of alternative imaging technologies. In the setting of liver transplantation sensitivity and specificity of these complementary imaging investigations have not been adequately investigated. A recent meta-analysis [9] showed excellent sensitivity and specificity for MRCP in comparison to ERCP; however, the authors concluded that there were significant design flaws in the studies included. Therefore, firm recommendation to establish the place of MRCP in this diagnostic algorithm is tenuous. While small studies of ERCP in liver transplant patients have shown it to be safe and provide not only real-time evaluation of the biliary tree but also requisite therapy [10, 11], there is limited data on patients evaluated for abnormal liver function tests and the role of diagnostic ERCP in this cohort of patients.

Given these prior limitations, we aimed to evaluate the frequency, yield, and safety of diagnostic ERCP in patients with liver transplant patients presenting with liver function test abnormalities. A secondary aim was to identify predictive factors that were significantly associated with therapy at the time of ERCP.

## 2. Methods

We reviewed all patients enrolled from January 2002 to June 2013 that were prospectively entered into our IRB approved ERCP database (number X030409001). Variables maintained in the database include demographics, indications, pre-ERCP investigations, procedure related details, and outcomes. Patients were followed up by an experienced nurse at 24 hours and 30 days with complications documented using consensus criteria [12]. Patients included were those who underwent ERCP for ductal evaluation and therapy for abnormal liver function tests. Patients were excluded if they had obvious biliary tract abnormalities and referred for therapeutic ERCP; patients with altered anatomy requiring percutaneous transhepatic cholangiography or double balloon ERCP examination and patients less than 18 years of age. Diagnostic ERCP was defined as procedure performed with the intent of diagnosis and requisite therapy. Therapeutic ERCP was defined as those requiring sphincterotomy, stone extraction, or stent insertion.

### 2.1. Statistical Analysis

The continuous variables such as age and the results of liver function test (LFT) were summarized as median and interquartile range or range. The categorical variables, such as demographic characteristic, prior image analysis, final diagnosis, therapy, and complication, were described as frequencies and proportions. The frequency of occurrence of abnormal LFT's result in orthotopic liver transplantation patients was determined with the denominator of the total number of patients who had ERCP and 95% confidence interval of population proportion was then calculated. Univariate logistic regression analyses were performed to assess the association between age, gender, biochemical, imaging results, and therapeutic ERCP. Variables that had *P* value < 0.1 and those judged to be clinically relevant were included in multivariate logistic regression model. A two-sided *P* value < 0.05 was considered significant. The datasets were compiled using Microsoft Access and SAS software, version 9.3 (SAS institute, Cary, NC, USA), was used to perform the analysis.

## 3. Results

**Figure 1:**
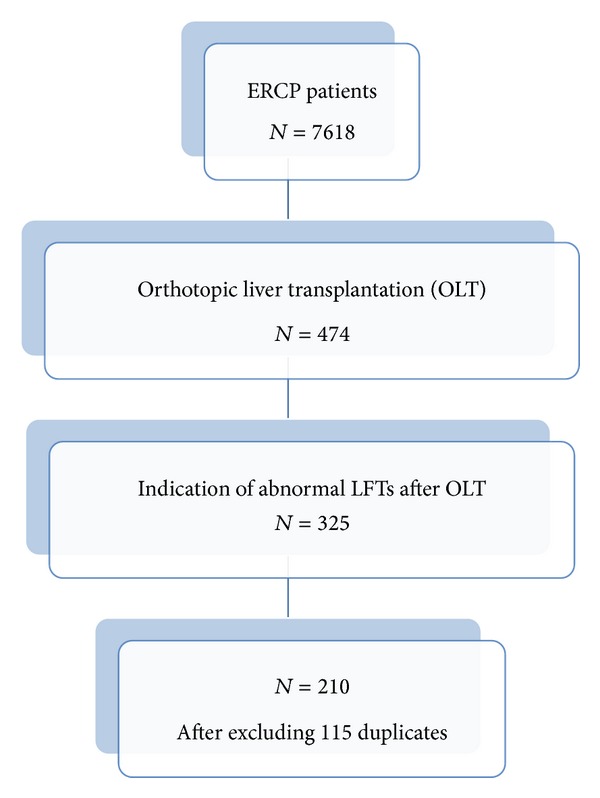
Flow chart of the patients accounted for in the study.

A total of 7618 ERCPs were performed between 1/1/2002 and 6/30/2013 and 474 ERCPs were performed on adult liver transplantation patients. After excluding repeated procedures, 210 patients (44.3%; 95% CI 39.8–48.8) were identified as having undergone index ERCP (Figure 1) for abnormal liver function tests in the setting of OLT; 83.8% were Caucasian and 62.4% were male with a median age of 55 years. All patients had at least one liver enzyme abnormality or elevated bilirubin (Table 1). Imaging showed isolated dilated bile ducts in 10.5% by transabdominal ultrasound and 17.6% by CT scan. No other abnormality was detected on imaging.

Biliary cannulation was successful in all patients with only one patient requiring precut fistulotomy for access. Findings included biliary stricture in 21%; isolated biliary dilation in 15%; choledocholithiasis in 11%; papillary stenosis in 4%; biliary leak in 2%; primary sclerosing cholangitis in 1%; and 1% with pancreatic cancer. Following diagnosis, 78 patients (37%) underwent biliary sphincterotomy; 74 (35%) biliary stent insertion; 26 (12%) stone extraction, and 27 (13%) balloon dilation of stricture, and no intervention was required in 43%. Complications of ERCP occurred in 6 patients including three (1.4%) with mild, self-limited pancreatitis, one (0.5%) with cholangitis and two (1%) with postsphincterotomy bleeding that was treated successfully with endotherapy (Table 2).

Univariate analysis of factors associated with therapeutic ERCP showed significant association with dilated bile ducts on imaging. Other factors evaluated did not show a statistical significance. Further multivariate regression analysis confirmed that abnormal CT scan (OR 10.07; 95% CI 3.49–29.05; *P* value < 0.0001) and ultrasound scan (OR 3.88; 95% CI 1.15-13.12; *P* value = 0.0290) were significantly associated with therapy (Tables 3 and 4).

## 4. Discussion

Biliary complications in OLT patients can be difficult to diagnose but are often suspected in patients who are found to have elevations of liver function tests. Traditional algorithmic approaches for investigation in posttransplant patients include transabdominal ultrasound, MRCP if the clinical suspicion or biliary tract complications are very high, or invasive procedures like ERCP and PTC. Transabdominal ultrasound examination has poor sensitivity and specificity for diagnosing biliary tract pathology but is imperative to rule out hepatic artery thrombosis, while the role of MRCP in this setting has not been well studied. This study is the largest to date examining the role of ERCP as a diagnostic modality for investigating abnormal liver functions tests in liver transplant patients. Our results suggest that diagnostic ERCP in this cohort of patients is safe and effective for both excluding biliary tract disease and providing endoscopic therapy.

Sanna et al. examined the safety and efficacy of ERCP in postliver transplant patients and showed a high technical success, clinical success rates, and low complications but patients included had not only abnormal liver function tests but also radiological changes indicating that the majority had ERCP with a therapeutic intent [10]. Similarly Elmunzer et al. examined the role of diagnostic ERCP in 86 procedures and concluded a yield rate of 66.3% compared to 56.7% in our study. However, in comparison with our cohort, the patient population included was heterogeneous with symptoms alone, those with abnormal liver function tests with negative radiology, and some with no prior imaging [11]. Moreover, in their study, the complication rates were higher at 10.5% in comparison to 6–9% reported by other studies of ERCP in liver transplant patients [13, 14]. Our study shows that ERCP in OLT patients was associated with a much acceptable rate of post-ERCP pancreatitis of 1.4% with no severe complications documented in any patient. Our data shows a much lower risk perhaps considering the significant proportion of diagnostic nature of the study cohort.

During the past decade, with the advent of high quality MRCP and endoscopic ultrasound (EUS), the value of diagnostic ERCP to investigate the extrahepatic biliary tree has been significantly reduced [15]. However, this assessment is in the setting of an intact biliary tree and such data cannot be extrapolated to the postoperative state with altered biliary ductal anatomy. EUS may be useful to examine choledocholithiasis but given the spectrum of diseases causing abnormal liver tests in these patients, this modality may be of less relevance. To our knowledge, there are no comparative studies of ERCP with MRCP to evaluate biliary tract pathology in OLT patients. With previous studies indicating the frequency of postoperative biliary stricture rate and the efficacy of ERCP with bile duct stenting [16], surgeons often prefer early ERCP to exclude this complication. Our results not only indicate a diagnostic yield of 57% but also provide the treating physician with a specific diagnosis. ERCP is seen as gold standard investigation because of the dynamic nature of cholangiography which makes assessment of the biliary tree more sensitive and accurate than other imaging modalities. This combined with safety shown by our data makes this a more attractive and potentially a cost effective choice.

There are several limitations in our study. This is a retrospective analysis of current practice from a single tertiary center and the results may not be generalizable to all centers. Our study lacks longitudinal followup; therefore the proportion of patients with a normal ERCP who had further biliary complications is unknown and the proportion of patients with organ rejection is not known. Thirdly, time from transplant to ERCP was not documented and, fourthly, known patient related factors that may contribute to post-ERCP pancreatitis such as immunosuppression medication use and renal failure were not recorded [13]. However, our study shows that the use of diagnostic ERCP in liver transplant patients with abnormal liver function tests is often therapeutic and successful with a low complication rate.

In conclusion, biliary complications remain a significant problem in liver transplant recipients. Our study shows that ERCP provides a safe diagnostic and therapeutic value in the management of patients with abnormal liver function tests. Further studies examining the role of MRCP and further predictive factors for therapeutic compared to diagnostic ERCP are warranted.

## Figures and Tables

**Table 1:** Demographics and laboratory investigations of patients entered into the study.

Age, median (IQR)	55 (48–62)
Sex, *N* (%)	
Female	79 (37.6)
Male	131 (62.4)
Race, *N* (%)	
Caucasian	176 (83.8)
African-American	30 (14.3)
Other	4 (1.9)
Liver function test, median (range)	
Total bilirubin	2.5 (0.2–36.5)
AST	78 (12–1375)
ALT	109 (9–1022)
Alkaline phosphatase	277 (51–1625)
GGT	339 (24–2050)

AST: aspartate aminotransferase; ALT: alanine aminotransferase; GGT: gamma glutamyl transferase.

**Table 2:** Therapy performed and complications after procedure.

Therapy, *N* (%)	
Precut	1 (0.5)
EBS	78 (37.1)
Stone extraction	26 (12.4)
Biliary stenting	74 (35.2)
Balloon dilation	27 (12.9)
Complications, *N* (%)	
Pancreatitis	3 (1.4)
Cholangitis	1 (0.5)
Bleeding	2 (1)
Perforation	—
Other	—

**Table 3:** Univariate analysis for therapeutic ERCP (*N* = 126, 60%).

	Odds ratio (OR)	95% CI for OR	*P* value
Age	1.01	[0.99, 1.03]	0.2914
Sex (male versus female)	0.87	[0.49, 1.55]	0.6418
Bilirubin (>2 versus ≤2)	0.06	[0.35, 1.20]	0.1668
ALT (>56 versus ≤56)	0.60	[0.29, 1.25]	0.1722
AST (>40 versus ≤40)	1.05	[0.47, 2.34]	0.8997
ALP (>118 versus ≤118)	1.56	[0.60, 4.07]	0.3606
GGT (>65 versus ≤65)	5.06	[0.94, 27.28]	0.0590
Ultrasound (abnormal versus normal)	3.76	[1.16, 12.16]	0.0272^†^
CT scan (abnormal versus normal)	10.72	[3.73, 30.80]	<0.0001^†^

^†^Significant result at 0.05 level of significance.

AST: aspartate aminotransferase; ALT: alanine aminotransferase; ALP: alkaline phosphatase; GGT: gamma glutamyl transferase; ct: computed tomography.

**Table 4:** Multivariate analysis for therapeutic ERCP (*N* = 126, 60%).

	Odds ratio (OR)	95% CI for OR	*P* value
Ultrasound (abnormal versus normal)	3.88	[1.15, 13.12]	0.0290^†^
CT scan (abnormal versus normal)	10.07	[3.49, 29.05]	<0.0001^†^

^†^Significant result at 0.05 level of significance.

CT: Computed Tomography.
